# Posttraumatic Stress Disorder and Mobile Health: App Investigation and Scoping Literature Review

**DOI:** 10.2196/mhealth.7318

**Published:** 2017-10-26

**Authors:** Carolina Rodriguez-Paras, Kathryn Tippey, Elaine Brown, Farzan Sasangohar, Suzannah Creech, Hye-Chung Kum, Mark Lawley, Justin K Benzer

**Affiliations:** ^1^ Department of Industrial and Systems Engineering Texas A&M University College Station, TX United States; ^2^ Center for Research and Innovation in Systems Safety Department of Anesthesiology Vanderbilt University Medical Center Nashville, TN United States; ^3^ Health Science Center School of Public Health Louisiana State University New Orleans, LA United States; ^4^ Center for Remote Health Technologies and Systems Texas A&M University College Station, TX United States; ^5^ Department of Environmental and Occupational Health School of Public Health Texas A&M University College Station, TX United States; ^6^ VISN 17 Center of Excellence for Research on Returning War Veterans Central Texas Veterans Health Care System Waco, TX United States; ^7^ Department of Health Policy and Management School of Public Health Texas A&M University College Station, TX United States; ^8^ Department of Psychiatry Dell Medical School University of Texas Austin, TX United States

**Keywords:** posttraumatic stress disorders, PTSD, mobile health, mHealth, anxietys

## Abstract

**Background:**

Posttraumatic stress disorder (PTSD) is a prevalent mental health issue among veterans. Access to PTSD treatment is influenced by geographic (ie, travel distance to facilities), temporal (ie, time delay between services), financial (ie, eligibility and cost of services), and cultural (ie, social stigma) barriers.

**Objective:**

The emergence of mobile health (mHealth) apps has the potential to bridge many of these access gaps by providing remote resources and monitoring that can offer discrete assistance to trauma survivors with PTSD and enhance patient-clinician relationships. In this study, we investigate the current mHealth capabilities relevant to PTSD.

**Methods:**

This study consists of two parts: (1) a review of publicly available PTSD apps designed to determine the availability of PTSD apps, which includes more detailed information about three dominant apps and (2) a scoping literature review performed using a systematic method to determine app usage and efforts toward validation of such mHealth apps. App usage relates to how the end users (eg, clinicians and patients) are interacting with the app, whereas validation is testing performed to ensure the app’s purpose and specifications are met.

**Results:**

The results suggest that though numerous apps have been developed to aid in the diagnosis and treatment of PTSD symptoms, few apps were designed to be integrated with clinical PTSD treatment, and minimal efforts have been made toward enhancing the usability and validation of PTSD apps.

**Conclusions:**

These findings expose the need for studies relating to the human factors evaluation of such tools, with the ultimate goal of increasing access to treatment and widening the app adoption rate for patients with PTSD.

## Introduction

### Mobile Health (mHealth) Apps

Recent technological advances have resulted in the development of emerging mobile health (mHealth) apps. mHealth apps include a wide range of applications such as educational materials and self-management platforms, health care–specific tools for managing the therapeutic process, health and preventative behavior, patient and patient-provider roles and relationships, challenges of daily life, and crisis situations. In addition, many of these apps are free and can help to reduce barriers to access, as they are able to provide the assistance to patients at any given time, whereas speaking with a clinician requires an appointment, traveling to the facility, and financial considerations. mHealth apps have thus far been successfully implemented across a wide range of medical disciplines, including dermatology [1], ophthalmology [2], and nutritional sciences [3] and have addressed many specific illnesses such as diabetes [4] and infectious diseases [5]. Apps have also been developed to aid in the treatment of mental disorders such as posttraumatic stress disorder (PTSD) [6-10].

### Posttraumatic Stress Disorder

PTSD is a prevalent mental health issue that commonly occurs after a person has experienced a traumatic event, which can include being threatened with death or experiencing the death of others (eg, death of a family member or a friend), sexual violence, or serious injury [11]. Approximately, 7.8% of the American population will experience PTSD at some point during their lifetime [12], with veterans being between 5% and 25% more likely to experience PTSD depending on the service era in which they served their country (ie, Vietnam War, Gulf War, Operations Iraqi Freedom, Enduring Freedom, and New Dawn) [11,13]. All diagnoses of PTSD must occur after a traumatic experience, but not all traumatic experiences lead to the development of PTSD. Diagnosis of PTSD involves symptoms from each of the following four clusters: (1) intrusion (eg, nightmares and flashbacks), (2) avoidance (eg, thoughts and feelings), (3) negative alterations to cognitions and mood, and (4) alterations to arousal and reactivity (eg, depression or sleep deprivation) [14]. As noted, military veterans are a special population whose warzone experiences may increase the risk for PTSD. Furthermore, many veterans diagnosed with PTSD still serve the military in active combat roles, with this number peaking at over 17,000 servicemen in 2012 [15]. However, evidence has supported the efficacy of multiple PTSD treatment options in reducing overall posttraumatic stress symptoms in veterans diagnosed with PTSD [16-18].

### PTSD Treatment

Current PTSD treatments are divided into two categories that are not mutually exclusive: (1) pharmacotherapy and (2) psychotherapy. Studies suggest psychotherapy is more effective than pharmacotherapy [16], with two cognitive behavioral therapy (CBT) treatment methods considered the most effective: (1) prolonged exposure (PE) therapy and (2) cognitive processing therapy (CPT) [16]. PE was developed from the idea of *prolonged* or *repeated* exposure to traumatic events [17]. Patients repeatedly encounter situations known to cause symptoms while in a safe environment, with the expectation that they will overcome their fears [17]. CPT helps patients understand and change the way they think about traumatic events by emphasizing that they are not at fault [18].

Despite the efficacy of current PTSD therapies, significant challenges still exist in access to treatment, particularly for veterans, that may influence both treatment-seeking behavior and adherence to treatment [19,20]. First, access to mental health care is becoming increasingly more difficult. Mental health facilities continue to be understaffed despite the actively growing demand for mental health care [21], with reports indicating that facilities serving veterans have an insufficient mental health workforce to meet the needs of personnel returning home from active duty [21,22]. Access to health care may be influenced by geographic, temporal, financial, cultural, and technological factors [23]. Geographic factors create difficulties in traveling to mental health facilities. Temporal factors include obstacles in scheduling care (eg, evening or weekend appointments). Financial factors center on the limitations of health insurance, co-pays, and other monetary resources needed to attend clinical sessions. Cultural factors stem from the societal stigma associated with receiving mental health care [24-26] and may be a particularly important factor in dissuading veterans from seeking care because of the military culture related to mental health care [27]. Finally, technological factors involve the different barriers in obtaining or using technologies.

mHealth apps have the potential to help improve access to care [28], foregoing some of the difficulties in accessing mental health care as stand-alone tools (the patient can use the app without a clinician) or tools used in coordination with the clinician or in conjunction with the clinical treatment. Apps may effectively address technological access challenges as smartphones are widely available (64% of Americans owned a smartphone in 2015) [29], and apps can make it easier for patients to access information about the diagnosis and treatment of mental disorders. Apps may address cultural access by offering patients a discreet mobile environment to manage their disorder. In addition, apps may lessen geographic access issues by decreasing the number of in-person appointments needed. A particular strength of apps is the potential to improve temporal access by allowing patients and providers to conduct some of their therapeutic work asynchronously. Patients can use apps to monitor and manage their symptoms, record and replay therapy sessions, connect with clinicians or emergency personnel in the case of crises [30], and engage in social connections with communities of trauma survivors for additional treatment support [31]. Clinicians may use apps to collect data on patient engagement and progress during and between sessions, such as how much time the patient spends doing practice assignments, how often patients utilize emotion regulation skills such as relaxation, and how well the treatment is helping to manage their symptoms. Along these same lines, the addition of external wearable sensors, such as those found in smartwatches, may provide both patients and clinicians with additional data on sleep quality and could potentially aid in determining events that trigger hyperarousal [7].

The goal of PTSD apps should be to aid in the treatment and monitoring of trauma survivors with PTSD and to provide both patients and clinicians with timely remote feedback that can supplement or enhance current therapies [10]. The potential benefits of properly validated PTSD apps are to effectively engage trauma survivors with PTSD, thus improving their access to care [32]. However, limited knowledge exists about the availability of apps for PTSD and about how well these apps were developed. The aim of this paper was to document the currently available PTSD-related apps as well as the usage of these apps and validation procedures used when designing them.

## Methods

### Overview of Research Methods

This study consists of two parts: (1) a review of publically available PTSD apps, which includes more detailed information about the three most prevalent apps used and (2) a scoping literature review performed using systematic methods. The purpose of part 1 is to determine the availability of PTSD apps, and the purpose of part 2 is to determine the usage and efforts toward validation of such mHealth apps.

### mHealth App Search Method

Health care providers’ mobile app websites (eg, the Department of Veterans Affairs [VA] App Store), commercial app stores (eg, Apple App Store, Google Play Store), websites that aggregated or listed mental health apps, websites that provide app ratings, Web communities supporting veterans, and Google were used to search keywords relating to [post-traumatic stress disorder” OR “PTSD”], [“veterans”], and words relating to PTSD treatment (eg, [“insomnia”]). The search was performed from January 2016 to August 2016. Data on available apps were collected from the following websites: Apple App Store [33], Google Play Store [34], VA App Store [35], National Center for Telehealth & Technology mobile applications site [36], and Amazon App store for Android [37]. All apps were available on at least one of these websites as of August 10, 2016. The following inclusion criteria was used: the app must be relevant to PTSD, PTSD treatment, or common symptoms of PTSD (ie, depression, anxiety, insomnia, and anger); treatment apps related to veteran support must be specific to the US military or US veterans; and the app had to be able to be used without opening a Web browser. Apps may include content related to mental health disorders that are comorbid with PTSD. For example, apps may address both PTSD and depression. However, no apps that solely addressed depression, and not PTSD, were included.

Apps were categorized according to clinical focus (ie, mental health disorder, PTSD symptom, or clinical treatment modality) and for each type of app utility used (eg, education and exercises). Each app was assigned to one primary clinical focus but could contain more than one app utility. For example, Acceptance and Commitment Therapy (ACT) Coach included mindfulness exercises, but the primary focus of this app is specific to a VA-recognized evidence-based treatment for PTSD. Apps may use several utilities. For example, PE Coach included multiple utilities such as educational materials and exercises. Apps within each clinical focus and app utility categories were tallied to determine frequency. The following information for each app was also collected to determine feasibility and acceptability: average user ratings, number of user ratings, availability to iPhone operating system (henceforth iOS) (Apple, Cupertino, CA) and/or Android (Google, Mountain View, CA) operating systems (henceforth Android), minimum iOS and/or Android requirements, and cost to download.

### Scoping Literature Review Method

The app search did not reveal information on how the apps were designed and evaluated, or whether studies had analyzed their usability. A scoping literature review [38] was conducted to further understand the scope of available evidence and support the efficacy of such tools.

A combination of keywords relating to [“post-traumatic stress disorder” OR “PTSD”] AND [“mobile applications” OR “mHealth”] was used to search within Google Scholar and the Texas A&M EBSCOHost Research Databases such as MEDLINE, ABI/INFORM Complete, and Academic Search Complete. The Google Scholar database search was completed on March 16, 2016, with a total of 1850 results. The search using other databases did not result in any new results. The search only included journals written in English and published in or after 2011, as this is the year in which PTSD Coach, the first of the VA PTSD mobile apps, first appeared on the app store. The following inclusion criteria were then used to narrow the scope of the papers obtained through the search: the paper reviewed or validated an existing PTSD app (eg, feasibility studies, randomized clinical trials, usability testing, etc), the paper detailed the development of a new app for the detection or treatment of PTSD, or the paper was a case study using PTSD apps.

## Results

The following section first details the results from the review of publically available PTSD apps and then the scoping literature review.

### mHealth App Search Results

A total of 201 apps were chosen for the study, all of which were available for iOS or Android. Apps were categorized based on their primary focus or purpose, which fell into six distinct groups. These were as follows: (1) PTSD evidence-based treatment (EBT), which included apps specific to a VA-recognized EBT for PTSD [18]; (2) PTSD-specific, which included apps that provided educational materials, exercises, and/or symptom tracking only for PTSD but were not specific to any evidence-based PTSD treatment method utilized at VA; (3) general mental health (MH) that included PTSD, which included educational materials and exercise content on multiple mental health conditions (eg, depression and anxiety), as well as content related to PTSD; (4) mindfulness and relaxation techniques commonly used with PTSD, which included content specific to mindfulness techniques or exercises; although these apps are not specific to PTSD or a treatment for PTSD, mindfulness techniques are a core component of ACT therapy, which is recognized at VA as an EBT for PTSD; (5) anger management, which is a common skills deficit in patients who have PTSD, and therefore, commonly addressed in treatment for PTSD; these apps were specific to anger management but were not specific to PTSD; (6) insomnia, another common symptom of PTSD, is also a frequent focus in treatment for PTSD; these apps were specific to addressing insomnia but were not specific to PTSD (Table 1).

The total number of 201 apps chosen included duplicate apps between operating systems; for example, PTSD Coach was available for both iOS and Android, so it was counted twice. When duplicates were removed, the total number of apps was 81. Across all categories, apps related to mindfulness and relaxation were the most frequently available (approximately 29.9%, 60/201) followed by PTSD-specific apps (approximately 22.4%, 45/201).

Apps were further analyzed for content and utilities, which included the following: (1) educational information, which included educational material regarding PTSD, PTSD treatment, or common symptoms of PTSD; (2) exercises, which included either skills training or practice components; (3) symptom tracking, which included tools to track severity of symptoms of PTSD, whether or not the app was related to PTSD (eg, tracking sleep); (4) connections to outside professional support, which provided methods of contacting outside professional support, and this included direct contact options (eg, send a message through the app) or providing contact information (eg, phone number); (5) connections to outside peer support, which included content for individuals with PTSD for communication with local or online peer support; and (6) components specific to treatment integration, which included content designed to be integrated into ongoing in-person treatment with a therapist. An example of treatment integration content included patients’ ability to audio-record in-person therapy sessions and replay them outside of therapy as part of a treatment homework assignment (Table 2). Tallies of app content categories that are included in Table 2 do not include duplicates between operating systems. For example, CPT Coach utilities were only tallied once and not twice for CPT Coach for iOS and for Android.

Across utilities, in-app exercises (eg, guided breathing) were the most commonly provided app function and were especially dominant across apps related to mindfulness and relaxation. All apps that were directly related to an EBT for PTSD included exercise components, and apps specific to PTSD commonly utilized both education and exercise components. Both PTSD-specific and mindfulness and relaxation app categories had at least one app to provide for one or more of all measured utility categories. All app categories included at least one app that provided education.

Building on this work, app accessibility was measured in two ways: (1) technological access, defined as the minimum operating system and memory required to use the app, with lower operating systems and less memory requirements having better accessibility and (2) financial access, defined as the cost of downloading the app, with lower costs having better accessibility. With respect to *technological access,* although some Android apps adjusted to the operating system on the user’s phone, several Android and iOS apps required relatively recent versions of operating systems (eg, iOS 8.1), which may act as a barrier to individuals with older smartphones with low storage space.

**Table 1:** The app categories grouped by the type of operating system.

Type of operating system	PTSD^a^ EBT^b^	PTSD-specific	MH variety	Mindful and relax	Anger	Insomnia	Total
iPhone operating system (iOS) only	8	12	8	22	4	12	66
Android only	7	14	5	16	4	8	54
Both iOS and Android	12	19	8	22	8	12	81
Total	27	45	21	60	16	32	201

^a^Posttraumatic stress disorder.

^b^Evidence-based treatment.

^c^MH: mental health.

**Table 2:** The app tallies for different utility categories (utilities are not mutually exclusive).

App categories (total from search)	Education	Exercises	Tracking	Professional support	Peer support	Treatment integration	Other
PTSD^a^ EBT^b^ (27)	5	12	4	2	0	2	2
PTSD-specific (45)	13	12	5	4	2	0	9
MH variety (21)	2	5	5	0	1	0	8
Mindful and relax (60)	7	22	7	2	3	0	19
Anger (16)	5	7	2	0	0	0	2
Insomnia (32)	3	11	3	0	1	0	14
Total	35	69	26	8	7	2	54

^a^Posttraumatic stress disorder.

^b^Evidence-based treatment.

For example, an Apple phone with 8 GB of space may not be able to download an upgrade that requires over 4 GB of available space. Regarding *financial access*, in 2013, a new smartphone cost, on average, US $531 in North America (Can $662.13, Aus $678.28) [39], and, for most major smartphone carriers, the first 1 to 3 GB of data costs an average of US $35 per month (Can $43.64, Aus $44.71) [40,41]. Most apps were either free or inexpensive to download, with the most expensive apps at US $9.99 (Can $12.46 , Aus $44.71). However, several apps included in-app purchases or equipment that cost much more than the app itself.

Three mHealth apps reviewed in this search appeared dominant among users (most downloads), all of which were developed by the Department of Veterans Affairs for trauma survivors with PTSD: PTSD Coach (261,045 total downloads), PE Coach (49,453 total downloads), and CPT Coach (11,689 total downloads). Download counts were reported by J Worthen from the National Center for Telehealth and Technology (September 12, 2016). These apps can be divided into two categories based on their intended use: (1) as a stand-alone app for the self-management of symptoms (ie, PTSD Coach) or (2) in conjunction with a PTSD EBT through a health care provider (ie, PE Coach and CPT Coach). PE Coach and CPT Coach are the only apps designed thus far explicitly for integration with standard treatment.

**Figure 1:**
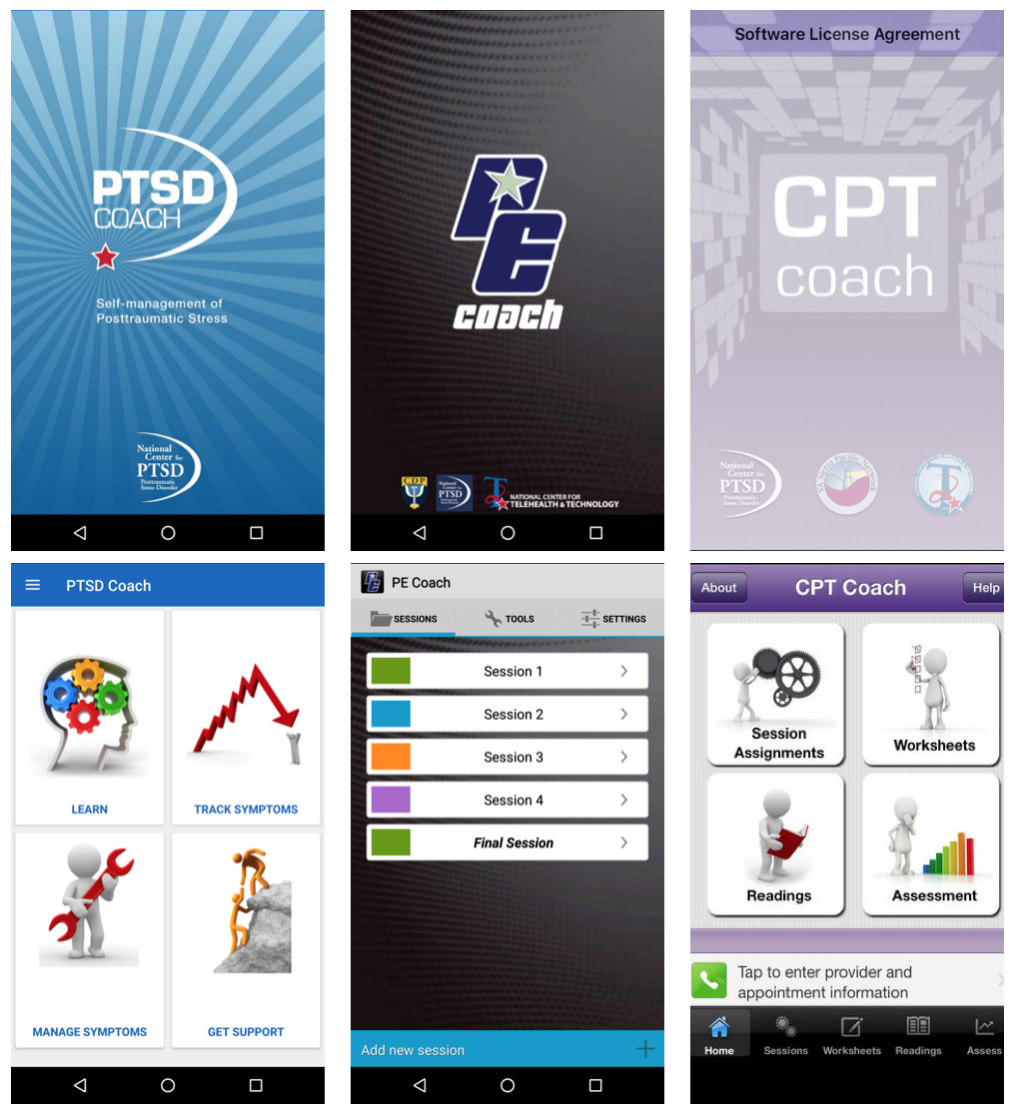
PTSD Coach (left), PE Coach (middle), CPT Coach (right).

PTSD Coach (Figure 1, left) is an mHealth app designed to explain PTSD concepts to patients and provide them with self-management tools based on CBT. Patients can use the tools in this app to learn, perform self-assessments, manage symptoms, and find support. The *learn* section provides trauma survivors and their family members with information about PTSD. The app’s *self-assessment* and *manage symptoms* sections provide individuals with a checklist to assess the severity of their symptoms, which patients can track over time to see their treatment progress and a list of mitigation techniques to cope with distressing situations, such as prompts to think about pleasant events and guidance through progressive muscle relaxation. After users finish using each mitigation technique, they are prompted to complete the checklist again; if the person rates their distress the same or higher, then they are offered another mitigation tool to try. The *find support* section allows trauma survivors to store contact information for those they rely on during emergency or crisis situations, making this information easy to access.

Of the 201 apps collected, only 2 were designed explicitly for integration with standard treatment for PTSD: PE Coach (with PE therapy; Figure 1, middle) and CPT Coach (with CPT; Figure 1, right). PE Coach is divided according to the different sessions for PE. For example, Session 1 contains only the PTSD Checklist assessment, the option to record the session, scheduling the next appointment, and the assigned homework, whereas Session 2 has the additional options to review previous homework assignments, add anchors for Subjective Units of Distress Scale, in vivo hierarchy, and in vivo homework, which occur only after the first session is complete. After Session 3, PE Coach also allows patients to record the imaginal exposure portion of the session, where the person processes the memory which causes their PTSD symptoms. Similarly, CPT Coach has information detailing the treatment components of CPT, homework assignments, and exercises. Both these apps also offer benefits to clinicians by allowing them to review a patient’s homework or monitor how much the patient is using the app during the session, allowing providers to see the patient’s between-session progress and to review these data with the patient during the session. In addition, the apps provide patients with reminders or help them schedule follow-up appointments.

### Scoping Literature Review Results

A total of 1850 papers were found. The 28 papers fitting the inclusion criteria are listed in Table 3. Table 3 also indicates whether or not each paper addresses some relevant usage categories identified in this research. The categories were defined to understand the human factors approach to the app design and development, including the population, any analysis performed on the app, the user demographics, and the usage and adoption. The categories were not mutually exclusive; thus, a paper could fit into more than one designation.

The first category “Veteran population” refers to papers that specifically mention PTSD apps in reference to the veteran populations (as opposed to the general population). “Benefit analysis” contains the papers that focus on the perception or evaluation of app, or with the potential benefits of apps to the population. “Age concern” includes the papers that discuss potential barriers to older populations, or the appeal of mHealth apps to the younger populations. “Usage and adoption” refers to papers that specifically mention how the users have interacted with the apps, particularly those that contain analytics on the use of apps, such as statistics about downloads, how often users return to the app after the initial download, and the number of users who download the app specifically as part of their treatment for PTSD. The final category “HFE considerations” (ie, human factors and ergonomics) contains the papers that mention app design concerns, including usability of apps, user satisfaction, and acceptability of apps, or other HFE analysis beyond that in the previous categories.

Although all the papers listed in Table 3 met the inclusion criteria, the majority only mentioned PTSD apps as an example or in passing. The 6 papers with the footnote in the reference column in Table 3 focused on more detailed analysis of a specific PTSD app—either PE Coach or PTSD Coach—discussing their usage or validation more extensively than the other papers and hence are discussed here in further detail.

Three studies examined PE Coach. Reger et al [44,60] and Kuhn et al [59] explored the functions and potential benefits of using PE Coach as an adjunct to traditional therapy. Studies conducted before the app’s release highlighted the features of the app [44] and surveyed clinicians’ perceptions on the usefulness of the app [59]. The only study performed after PE Coach’s release was Reger et al [60], which was a case study examining two soldiers’ perceptions of the usability and their satisfaction with the app when implemented along with PE therapy. Although all these studies had positive results, most noted the need for additional testing to determine the app’s impact on clinical outcomes.

Three studies examined PTSD Coach, all of which were more analytical than those that examined PE Coach. Both Kuhn et al [9] and Owen et al [42] explored app usage, with Kuhn et al [9] focusing more on subjective perceptions, such as user satisfaction and helpfulness, and Owen et al [42] focusing more on objective user engagement via Flurry Analytics software (Yahoo! Developer Network). Owen et al [42] was also able to determine common points of attrition in using the app. The final paper [46] was a randomized control trial evaluating the effectiveness of PTSD Coach as a stand-alone app versus when used in conjunction with clinical support; both treatment groups had clinically significant improvements in their symptoms but having clinician support resulted in greater reductions in PTSD symptoms.

**Table 3:** The 28 papers meeting the inclusion criteria. The headers are the categories of gaps identified. An “X” in the column indicates that the papers worked to address this gap.

Paper number and author name		Veteran population	Benefit analysis	Age concern	Usage and adoption	HFE^a^ considerations
1	Erbes et al [6]	X	X	X	X	X
2	Gravenhorst et al [7]			X		X
3	Chen et al [8]	X				
4	Kuhn et al [9]^b^	X	X	X	X	X
5	Olff [10]					
6	Sloan et al [30]	X				
7	Owen et al [42]^b^	X	X		X	X
8	Kuhn et al [43]		X	X		X
9	Reger et al [44]^b^	X				
10	McInnes et al [45]	X		X		X
11	Possemato et al [46]^b^	X	X			
12	Gratzer et al [47]					
13	Kuester et al [48]					
14	Luxton et al [49]					
15	Fletcher et al [50]		X			
16	Castro et al [51]	X				
17	Turvey et al [52]					
18	Kanuri et al [53]					X
19	Baysari et al [54]					
20	Mohsenin et al [55]					
21	Driesenga et al [56]	X				
22	Proudfoot [57]					
23	Price et al [58]	X				
24	Kuhn et al [59]^b^		X	X		X
25	Reger et al [60]^b^	X	X			X
26	Olff et al [61]		X			X
27	Weingardt and Greene [62]	X				
28	Chan et al [32]					

^a^Human factors and ergonomics.

^b^Papers that provide more detailed analysis of a specific PTSD App (PE Coach or PTSD Coach).

To date, no papers exist that compare the stand-alone app (PTSD Coach) with the adjunctive apps (CPT Coach and PE Coach). Although PTSD Coach offers more information on PTSD and some therapy tools, both PE and CPT Coach contain information relevant for each clinical session. For example, these adjunctive apps may be able to improve treatment adherence by offering reminders on homework assignments due. Clinicians’ perceptions tend to be favorable toward implementing apps for PTSD treatment, such as PE Coach [43]. Similar apps that address mental or chronic conditions include those for helping users manage stress [63] and for aiding arthritis patients in medication and exercise compliance [64].

Some of this literature conceptualizes mHealth apps as a method for overcoming geographic and temporal access barriers to mental health care [42]. Although there are few papers evaluating the PTSD apps’ effectiveness, the papers reviewed indicated that mobile phones may be an adequate supplement for PTSD treatment [7]. Increasing temporal access through mHealth apps to health services may also result in lower cost, improved patient satisfaction, and improved health outcomes [8].

## Discussion

### Principal Findings

The goal of this review was to determine the availability and level of validation of PTSD apps. The mHealth app search indicated that a plethora of Android and iOS PTSD-specific apps are available. In addition, the app search showed that other PTSD-related apps such as relaxation, insomnia management, and anger management tools are available but may not be necessarily known to PTSD patients. Despite the availability of these tools, the scoping literature review suggested there was insufficient evidence on the validity of the apps. The literature review highlighted the need for additional studies on app dissemination and adoption [6,32,44,45,49,53] and app validation and treatment integration [7,43,44,46-48,52,59]. This literature also suggests the potential of integrating such PTSD-related apps with new technologies, such as smartwatches, as an adjunct for improving treatment.

Of the PTSD-related apps available, all of the VA’s apps including the three most-downloaded apps for PTSD treatment (PTSD Coach, CPT Coach, and PE Coach) are free to download. However, the results suggest many trauma survivors with PTSD, and even some practitioners, may not be aware that these apps exist [8,6,10,52,57]. Many factors may influence app adoption, including social contacts [65]. In addition, there are different business models that can be implemented depending on the type of app [66]. Additional concerns over app usage remain, focusing on issues relating to patient data confidentiality, data storage, legal and ethical issues [67], and on issues relating to the cost of smartphones and data plans, app software requirements, and the cost of some apps developed by non-VA developers. More information is hence needed on the dissemination and adoption of these mHealth tools and how to improve their accessibility to PTSD patients.

This review found only 6 papers analyzing the usage or validation of specific PTSD-related apps [9,42,44,46,59,60]. Of the 6 papers examined in further detail, none provided evidence of the implementation of user-centered procedures during the app development to improve usability. High-level usability fosters user engagement and is essential to ensure such apps reach a broad audience of veterans that include those with PTSD receiving minimal or insufficient treatment [68]. Evidence also suggests the majority of the PTSD-related apps have yet to be validated, with no validation efforts reported for these apps in the patient-provider context before their release [44]. These papers also highlighted the small sample sizes in user studies conducted postapp release [9,31,60]. This lack of documentation of the user-centered design and testing process is less rigorous compared with traditional validation procedures set out by the US Food and Drug Administration. An important question to ask is what should be the level of validation needed for these apps. App validation will incur into additional time and monetary resources for app developers. However, the lack of validation data limits the ability of consumers to determine which apps may be helpful in managing PTSD symptoms. A good balance might be to conduct selective postrelease validation studies on popular apps.

The potential legal ramifications posed by mHealth apps center on scrutiny of data storage and remote communication with health care providers. The responsible party for these legal issues—whether it is the developer, the health care provider, the user, or some combination of the aforementioned—has yet to be determined [7]. Current PTSD-related apps reside solely on the smartphone and involve no data communication with any hospital system. The threat of legal ramifications for data security issues relating to remote communication is the primary reason why many PTSD-related apps do not have user accounts or remote connections with clinicians [8]. Data synchronization issues within complex health care environments and accountability after automatic data notifications contribute to health care providers’ concerns.

Despite potential security issues and obstacles to apps’ dispersion among users, an abundance of calls for more studies on app dissemination and adoption [6,32,44,45,47,49,53] and on app validation and treatment integration [7,43,44,46-48,52,59] indicates the importance of developing apps with optimal usability and improve treatment. Of those apps designed to be used in conjunction with clinical treatment, only two currently incorporate specific treatment-related components (PE Coach and CPT Coach). This indicates a need for further app development and refinement to include treatment integration components such as objective measures of PTSD. These objective measures may be provided through adding functions incorporating wearable sensors into PTSD-related apps, which would provide valuable information to aid in treatment.

Recent advances in wearable sensors provide an opportunity for future PTSD apps to utilize the features of activity trackers for tracking patients’ PTSD-related physiological changes, thus aiding in diagnosing, monitoring, and optimizing clinical treatment regimens. This has the potential to significantly contribute to on-going therapies. App-integrated information from wearable sensors could be used to inform patients of their current physiology and mental state or provide clinicians with information about the most viable treatment course or aid in remote care [54]. Voice modulation has successfully been used to identify depression and anxiety, indicating its potential to detect PTSD triggers [69]. Physiological monitors, such as heart rate straps and watches, can also connect to mobile apps and act as an indicator of PTSD trigger [50,70], particularly because trauma survivors with PTSD tend to have a higher heart rate variability, which is an autonomic indicator of how they cope with stress that is currently being independently tested for biofeedback therapy [70].

The mHealth app search centered around four main symptoms of PTSD (eg, intrusion, avoidance, negative alterations to mood and cognition, and changes in arousal and reactivity), which are common among mental health issues including PTSD. Apps specific to other mental conditions that may aid in PTSD were hence not included in this analysis. In addition, although we focused on stand-alone mobile apps, there are several mobile-responsive websites related to PTSD that were not included in this search. Although accessibility was assessed and discussed based on technical and financial access, future studies should investigate design for the disabled (eg, blind, cognitively impaired, deaf, and users with missing limbs). Although the literature review yielded only a small number of peer-reviewed publications relating to PTSD mobile apps, the combined results of the app search and review of literature shed light on the current state of mHealth apps to support PTSD patients.

### Conclusions

This dual review highlights the availability and potential of PTSD app usage in increasing treatment adherence and quality. Results of this review suggest that current app development, however, lacks strong usability and validation components and that not enough apps are being developed to be integrated as treatment tools. These findings expose the need for studies relating to the human factors evaluation of such tools, with the ultimate goal of increasing access to treatment and widening the app adoption rate, for patients with PTSD.

## Abbreviations

ACT: Acceptance and Commitment Therapy
CBT: cognitive behavioral therapy
CPT: cognitive processing therapy
EBT: evidence-based treatment
HFE: human factors and ergonomics
mHealth: mobile health
PE: prolonged exposure therapy
PTSD: posttraumatic stress disorder
VA: Veterans Affairs
